# Regulation and state capacity

**DOI:** 10.1177/10434631221130850

**Published:** 2022-09-30

**Authors:** Arjun Chowdhury

**Affiliations:** 8166 University of British Columbia, Canada

**Keywords:** State capacity, regulation, rent-seeking, neo-patrimonialism, witchcraft

## Abstract

While one might expect states with low capacity to regulate less than states with high capacity, this is not supported by evidence, leaving open the possibility of rent-seeking. I use the example of the regulation of witchcraft in parts of Africa to informally model the conditions under which states with low capacity still come to promulgate a range of regulations even in the absence of rent-seeking interests. The model suggests that regulation can be a substitute for basic state functions like policing. I identify one normatively troubling aspect of this; the conditions under which such regulation might still improve state capacity over time, which qualifies claims made about rent-seeking and neo-patrimonialism; the model’s implications for contemporary state formation; and the parallels between the regulation of witchcraft and the regulation of offensive speech.

What is the relationship between state capacity and regulation? Two influential public choice explanations suggest a positive relationship. Functionally, more powerful states might be expected to regulate more because they can more easily fulfil basic functions: the supply of regulation is higher (Besley and Persson, 2009). Cynically, rent-seeking incentives should be greater in more powerful states: the demand for regulation is higher (Olson, 1982, 74). An adjacent explanation focusing on rent-seeking or neo-patrimonalism in lower-capacity states still identifies the existence of organized interest groups such as urban workers as the motive for regulation (Bates, 1981, 74–77). For the most part, states with fewer resources to fulfil basic functions like law and order and collecting taxes might be expected to first fulfil these tasks and then regulate other aspects of life. When lower capacity states do regulate excessively, it is likely because they face sectoral demands.

These logics would surprise citizens of lower capacity states who spend countless hours negotiating bureaucracies and proffering documentation which appears to serve no purpose. This has been labelled ‘government of paper’ by one anthropologist of Pakistan (Hull, 2012). Of the proliferation of documentation within the Indian bureaucracy, another observed, “the vast majority of bureaucratic writing is never read, even by other bureaucrats whose job it is to monitor subordinates… it is a form of production without a consumer” (Gupta, 2012: 152). More prosaically, price controls on food and energy are more prevalent in poorer countries, which tax proportionately less, than they are in wealthier ones (World Bank, 2020: 51–54). In Appendix 1, I plot four measures of regulation – the number of procedures to open a business or get a construction permit, and the time it takes to open a business or get a permit – against three measures of state capacity – tax ratios, immunization rates, and census accuracy. In *none* of the 12 plots do states with less capacity regulate any less than states with more capacity. To counter the claim that such regulation is explained by rent-seeking, I must identify a situation where regulation develops in a context of low state capacity and no rent-seeking.

To lay out how this can be, consider two premises underpinning the rent-seeking argument: an organized interest must exist, which is obvious, but, less obviously, so must an activity that can be regulated. Price controls on food, for example, presume organized urban workers who would benefit from the imposition of the control, and a transaction on which the control can be imposed. I leverage the second premise, that rent-seeking presumes the existence of an activity that can be regulated, to pose a seemingly strange puzzle: under what conditions does a government come to regulate an activity that does not exist, or at the very least, whose existence cannot be verified?

This seems absurd. But one can identify an activity whose existence cannot be verified, yet is regulated in low-capacity states: witchcraft in several countries in sub-Saharan Africa. By witchcraft, I mean acts of “occult violence” or “harm by uncanny means” where an individual alleges another, a so-called witch, has cast a spell that has inflicted harm (Ashforth, 2005; Hutton, 2017: 10). Witchcraft defies standards of verification common in criminal and medical contexts: “in instances of alleged occult practices … it is usually impossible to establish a direct link between cause and effect – which is what makes it occult in the first place” (Comaroff and Comaroff, 2004: 199). The analytical leverage the case of witchcraft offers is that because the act of witchcraft is unverifiable, there cannot be an organized interest that benefits from its regulation. The regulation of witchcraft in sub-Saharan Africa presents a limiting, or ‘hard,’ case for regulation because it is low on the values of the supply and demand variables – state capacity and rent-seeking – we would expect to drive regulation. I leverage the epistemological problem posed by witchcraft to provide a simple model for the expansion of regulation in the absence of rent-seeking.

To summarize: witchcraft itself is unverifiable, but individuals make verifiable accusations against alleged witches and inflict verifiable violence against alleged witches in sub-Saharan Africa, so constituting a limited but non-trivial problem of public order.^1^ Governments might be expected to respond by enforcing laws against murder or the infliction of bodily harm, but they prefer to regulate witchcraft when the cost of enforcing basic laws exceeds the cost of regulating witchcraft, and the regulation of witchcraft improves public order to a degree greater than the cost of regulation. The problem is that even if public order is improved by the regulation – citizens reduce their violence against witches – the regulation imposes costs on the government and alleged witches instead of individuals who would harm witches. While this is normatively troubling, I also analyze other, possibly more salutary effects of the regulation of witchcraft. This has implications for our understanding of neo-patrimonialism and state formation. To emphasize, the paper is not about witchcraft per se; it aims to theorize the process through which states expand regulation, despite being putatively ‘weak,’ in much of the world.

The paper is structured as follows. The first section describes the problem reactions to witchcraft pose to public order in contemporary Africa, efforts to regulate it, and addresses alternative explanations for such regulation. The second section lays out an informal model of the interaction between the government choosing whether or not to regulate witchcraft, and the citizens choosing whether or not to harm the witch. The third section extends the model to see if the regulation of witchcraft, despite its problems, can have salutary effects, which qualifies some of the claims made by scholars of neo-patrimonialism. The fourth section expands the analysis to the regulation of offensive speech. A final section concludes.

## Witchcraft and its regulation as empirical phenomenon

Before describing witchcraft beliefs and regulation thereof in Sub-Saharan Africa, I note that state capacity in that region is generally lower than elsewhere. Tax revenue, for example, is about half what it is in OECD states as a proportion of GDP (Keen and Mansour, 2009). These gaps are not new: two decades ago African states had proportionately fewer public servants than Asian or Latin American states (Goldsmith, 2000: 5–6). A measure of relative vulnerability to political and economic crises (‘state fragility’) identifies 13 African states among the 20 most vulnerable states, and no African states among the 20 least vulnerable (Fund for Peace, 2019). At an everyday level, over a third of respondents in the latest Afrobarometer survey said they did not feel safe walking in their neighborhood, and almost 10% of respondents reported being physically attacked in the past year (2016/2018, Round 7). For comparison, consider that in the US, 2% of individuals over 12 report being ‘violently victimized’ (Bureau of Justice Statistics, 2018). With low tax receipts and high crime rates, sub-Saharan African states would seem to be ill-equipped or ill-advised to expend resources to regulate witchcraft. But, as I describe now, this is in fact what they do.

Despite significant variation in the region, witchcraft beliefs are widely held. Two surveys suggest that in most of the countries surveyed a third (Pew Forum, 2010: 178–182) to half (Tortora, 2010) of respondents believed in witchcraft.^2^ Responses to witchcraft are a violent, but disaggregated phenomenon, because witchcraft is treated as a mostly private matter conducted among intimates (Geschiere, 2013). As such, witchcraft rarely constitutes a political threat to the government that would necessitate a response, but nor is it benign for public order.

It is important to clarify the verifiable from the unverifiable in acts of occult violence because the prospect of government action hinges on this distinction. The act of witchcraft is unverifiable: the government cannot establish that a spell produced harm in the way that it can observe a speeding car strike a pedestrian and react to it. But the accusation of an act of witchcraft *is* verifiable, and so are violent responses to such unverifiable acts. To the degree the government responds to an act of witchcraft, it responds to the (verifiable) allegation of the act, and/or the (also verifiable) response to it. Most African governments – the exceptions are Uganda and Zimbabwe – have not acknowledged the existence of witchcraft *even those that regulate witchcraft*. This contradiction was noted by a quasi-legal body in Zambia that urged that country’s Witchcraft Act, which punishes alleged witches with fines and up to 2 years of imprisonment, be amended to “acknowledge the existence of Witchcraft in Zambia” and “provide a clear definition of witchcraft” (Zambia Law Development Commission, 2018: 13).

At the extreme of verifiable reactions, alleged witches have been subject to stigmatization and violence. In Tanzania, hundreds of alleged witches have been murdered (Legal and Human Rights Centre, 2018: 18). In northern Ghana, nearly a thousand alleged witches were banished from their villages to so-called ‘witch camps’ (Action Aid, 2012). Almost 10% of 1300 human rights violations, including murder and torture, recorded by the UN in the Central African Republic in 2015 and 2016 were committed against alleged witches (United Nations, 2016: 18–19). Across West Africa two decades ago, in response to an unprecedented urban phenomenon called ‘penis-snatching’ – or the theft of one’s genitals upon a handshake from a stranger – crowds lynched dozens of suspended offenders (Bonhomme, 2016). In the Democratic Republic of Congo, hundreds of children have been abandoned on suspicion of being witches (Human Rights Watch, 2006: 47–49). In northern South Africa in the late 1980s, hundreds of alleged witches were killed, often by ANC cadres (Harnischfeger, 2000; Niehaus, 2001). In many cases, accused witches are predominantly female, and older (e.g. Miguel, 2005; Action Aid, 2012). Caveats are in order: within countries and over time, the level of violence against witches has varied significantly. And the idiom in which witchcraft and witchcraft accusations emerge is dependent on local conditions and changes therein; for example, the rise of Pentecostalism has been associated with greater attention to witches (Meyer, 1998). Witchcraft and the reaction to it in contemporary Africa is linked to historical beliefs, but is also affected by recent events and varies over time.

One can find assertions that violence against alleged witches has increased in the last four decades (Ashforth, 2015: 5–7). However, available data is neither standardized nor adjusted for population growth, so comparison over time and to other sorts of persecution needs to be treated with caution (Forsyth, 2016). I sought to first establish the highest reported level of reported violence in any country relative to population over time. I did not find any country where the deaths in any year were reliably reported as approaching 1000. The only longitudinal data is from Tanzania, where witch-killing averaged 250 a year between 1970 and 1988, at peak 1.8 deaths per 100,000 people, but much higher as a proportion of older women (Miguel, 2005). Tanzania appears to have had the highest level of sustained persecution of witches (as well as the highest reported levels of beliefs in witchcraft). Recent allegations of witch-killing in Tanzania of 300–500 witches a year between 2014 and 2017 – around 7–10% of total homicides in that country – is slightly below the levels measured in the 1970s and 1980s when adjusted for population growth (<1 death/100,000 people).

Elsewhere the scale of violence appears to be in the dozens rather than hundreds of deaths per year, although there are occasional spikes, such as in northern South Africa in the late 1980s and early 1990s. To give a comparative sense, 4000 African-Americans were lynched between 1877 and 1950 in the United States. Lynching was not uniform over time; it peaked at 1.9 deaths per 100,000 African-Americans in 1892 (higher for African-American men).^3^ The highest level of witch-killing in any country in Africa would appear to be somewhat similar to the highest level of lynching in the southern United States but these ratios should be taken advisedly as both lynching and persecutions of witches are socially, geographically and temporally concentrated, meaning the appropriate denominator is not obvious.

Governments have responded to both witchcraft and witch-killing with a variety of measures to punish witches, but mostly without legal acknowledgment of the existence of witchcraft.^4^ These are not new – state-organized campaigns targeted alleged witches in the 1970s in Benin, Malawi, and Zambia (Behringer, 2004: 11–12, 211). More recently, in the Gambia, where there are no laws pertaining to witchcraft, government forces were alleged to have detained hundreds of witches (Amnesty International, 2009). Malawi does not ban witchcraft but proscribes its pretense, and dozens of alleged witches have been imprisoned there (Smith, 2010). In Mozambique, the FRELIMO government initially dismissed beliefs in witchcraft (known as ‘sorcery’), but has recently changed its approach, allowing local cadres to mediate in disputes over witchcraft accusations when previously the government would have punished those making those accusations (West, 2005). Several national and local governments have laws on the books that have been used to punish both individuals who represent themselves as witches or engage in witchcraft, and those who persecute alleged witches, including Nigeria, Kenya, and Zambia. Variants of these laws can be found in both former British and French colonies, including Nigeria, Kenya, Zambia, Cameroon and the CAR.

Some of these prohibitions date from the colonial era, during which, in British colonies, the existence of witchcraft was denied and accusations of witchcraft were punished, not witchcraft itself (e.g. Waller, 2003). With few exceptions (e.g. Burkina Faso), these colonial-era legislations have not been entirely repealed. Reviews of these legislations and suggested reforms, for the most part, call for altered regulation of witchcraft. For example, the 1996 Ralushai Commission in South Africa advocated repealing the apartheid-era Suppression of Witchcraft Act and replacing it with legislation that would “control” witchcraft by accepting the testimony of traditional healers in courts, and bureaucratizing this profession on the model of the Traditional Healers Association of Zimbabwe (Hund, 2000: 385–386). Later, when the Suppression of Witchcraft Act was struck down as unconstitutional by the South African Supreme Court, the Court still called for regulation of “harmful witchcraft practices” on the grounds that they could cause “intimidation with the intent to cause psychological distress and terror” and judges have reduced sentences because beliefs in witchcraft are seen as mitigating factors (Comaroff and Comaroff, 2004: 193–195). In Tanzania, the 1992 Nyalali Commission that recommended liberalizing measures noted the colonial provenance of the 1922 Witchcraft Ordinance and recommended it be repealed. But the subsequent 2002 Witchcraft Act still declared illegal anyone representing themselves as a witch or accusing another of being a witch; the only concession to Nyalali-style reforms was allowing District Commissioners to relocate accused witches if they were satisfied that they were either causing harm or “practicing witchcraft for gain or reward.” Only Uganda and Zimbabwe recognize the existence of witchcraft.

This non-systematic collection of evidence allows me to discount three alternative explanations for the development of recent witchcraft regulation. First, regulation might result from lobbying by organized groups like churches and medical associations. Because I cannot prove an absence, I cannot rule this out, but most of the anthropological studies suggest that public responses to witchcraft are not especially organized (Bonhomme, 2013). Witchcraft is a potent motif in many Christian, especially Pentecostal, congregations, but these groups generally combat witchcraft through their own rituals rather than demand government action (Meyer, 1998). Similarly, there is little evidence of medical groups lobbying for the acceptance of evidence from witches. When a medical group has opined on such topics, the body, Doctors for Life in South Africa, filed a case *opposing* the government’s effort to recognize traditional healers because their cures were not verified or efficacious.

Second, diffusion of international norms does not seem to be a significant factor in the development of these regulations. I could not find any endorsement of witchcraft regulation by any international organization, including the African Union. When United Nations agencies discuss the subject, as in the Central African Republic instance discussed above, it is generally to criticize the persecution of witches by individuals and governments. In any case, norm diffusion should lead to more uniform regulation rather than the variety of approaches described above. The wide variation in witchcraft beliefs within Africa, not international norms, likely accounts for much of the policy variation within the continent, because these beliefs vary much more than state capacity does (in any event, even higher capacity states like South Africa have proven incapable of prosecuting witch-killers, suggesting the upper bound on capacity is relatively low).^5^

Third, governments might regulate witchcraft because of regime insecurity. Certainly, opposition to witchcraft has provoked collective protest on occasion (e.g. Redding, 1996). However, collective protest is the exception; responses to witchcraft are more commonly localized instances of violence (Geschiere, 2013). Even when the incidents are frequent, they are rarely coordinated by a regime opponent. An anthropologist described ‘anti-witchcraft movements’ as having “little formal organization,” relying on personalist qualities like charisma, and thus being of “transient and recurrent nature” (Bonhomme, 2013). This is not to say that regime insecurity can never lead a government into increasing the scope of regulation: post-Soviet states, for example, regulate the cost of funerals to pre-empt protests about inflation (Sadigov, 2021). But this does not seem to be why African governments regulate witchcraft.

The essential point is that many governments in Africa are regulating witchcraft despite acknowledging that witchcraft cannot be verified using the standards of legal proof that underpin regulation, and without much evidence of organized interests clamoring for this regulation. This was epitomized by a Cameroonian case in which a judge, finding a woman guilty of rendering her lover impotent (with all women except herself), stated that while witchcraft could not be established scientifically, he nevertheless decided the defendant’s guilt based on his “firm convictions” (Fisiy, 1998: 157).^6^ In northern South Africa, the police indicted a healer for abetting a homicide by smearing the alleged suspects with goat’s blood to render them invisible. The prosecutor was less confident, stating, “it is going to be very interesting … to see how the courts handle evidence on whether the ritual to make the boys invisible was effective” (quoted in Comaroff and Comaroff, 2004: 200).

These evidentiary problems notwithstanding, witches face costs if found guilty. In the aforementioned case in Cameroon, the guilty party was sentenced to 8 years in jail and a significant fine, a judgment upheld by the Court of Appeal. When the government regulates witchcraft, it incurs costs of verification and enforcement, and imposes costs on the witch. But even though African governments regulate witchcraft, they do so ambivalently and with difficulty. This suggests the need to theorize the conditions under which governments regulate witchcraft, an activity whose existence they do not acknowledge.

## Regulating the unverifiable

Even though the existence of witchcraft cannot be verified, harms done to witches can be verified. The obvious answer to the question of why governments would regulate (unverifiable) witchcraft is that by doing so, they reduce (verifiable) harms inflicted on witches and to public order more generally. But this begs the question of why governments cannot achieve the same goals by enforcing laws to punish the infliction of (verifiable) harms inflicted on witches.

A more complete answer would be that the regulation of witchcraft improves public order at a lower cost than enforcing laws against homicide. This might be because the level of witch-killing is so high that the police are overwhelmed, or because cracking down on witch-killers provokes challenges to government officials, as occurred in Tanzania in the 1970s (Abrahams, 1987). Regulation must dissuade at least some citizens from harming witches, otherwise it is moot. For the government, the cost of regulating witchcraft must be lower than the value of the improvement in public order. This is straightforward, but the implication is troubling. Essentially a government is punishing alleged witches – recall these are individuals whose ‘crimes’ cannot be verified by normal legal standards – to prevent others from inflicting even greater, and observable, harm on them and otherwise disturbing the peace. The paradox of effective regulation of witchcraft is that the government is punishing witches to protect them (Ashforth, 2015: 30). As an NGO worker in the CAR said, “if we do not apply laws against PCS (practice of charlatanism or sorcery), we will apply *lex talionis* (an eye for an eye)” (Wood, 2010). More formally, the government is imposing a cost on the witch which is less than the cost she would face if the citizen punished her instead of the government, at some cost of enforcement to the government. Because the government prefers to punish the witch to prevent harm to the witch than punish an individual who would harm the witch, the latter is receiving a benefit *for not engaging in criminal activity*. Even if this is less costly for the government than enforcing basic laws, and less costly for the witch than being harmed, regulating witchcraft involves a transfer that is normatively troubling.

**Figure 1.**
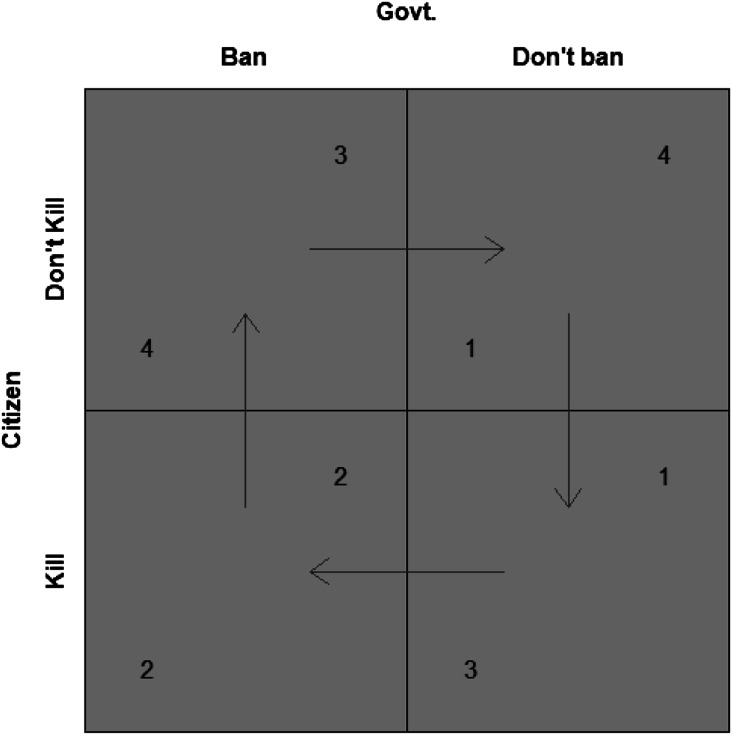
Regulating the unverifiable.

One can represent these trade-offs through a simple model where the government regulates witchcraft to prevent harm done to witches as shown in Figure 1 below. The government can choose to regulate or not regulate witchcraft, and the citizen can choose to harm or not harm the witch. I have labelled regulation as ‘ban’ and harm witches as ‘kill.’ The values in the bottom left of each cell are the citizen’s payoffs, those on the top right are the government’s. The highest value is denoted by 4, and the lowest by 1. The arrows represent best responses given what the other player does; for example, when the Government plays ‘ban,’ the Citizen plays ‘don’t kill’ and the arrow runs from the lower to the higher payoff.^7^

A significant feature of this setup is that the government’s preferences for regulation are different from the regulation of harms that can be verified and that cost more than the cost of regulating them. The government can be expected to prefer regulation over not regulating when it comes to activities that produce verifiable harms; even if the murder rate drops to zero, the government will not stop regulating murders, for example.^8^ Witchcraft is different: African governments have regulated it with some ambivalence, e.g. not acknowledging the existence of witchcraft. Because of this epistemological problem, the target of regulation is not witchcraft itself, it is the reaction to witchcraft, which is. Once citizens refrain from harming witches – bringing down the observable costs of witch-harming relative to the cost of the regulation plus the cost of punishment for the witch – the government prefers to cease enforcing the regulation. Because witchcraft itself, if not the response to it, is unverifiable, there is no equilibrium in pure strategies: the government would prefer not to regulate an unverifiable activity if citizens do not disturb public order in reaction to it, and citizens have to disturb public order in order to get the government to regulate it.^9^ This is the analytical leverage provided by the unverifiable aspect of witchcraft. Unlike verifiable harms, where the government prefers to regulate regardless of whether citizens react adversely or not, when it comes to witchcraft, the government should only regulate in response to adverse reactions by citizens.^10^

There is no equilibrium in pure strategies in this model. The only equilibrium is in mixed strategies (with these payoffs, the government bans witchcraft with probability 0.5, and the citizen kills the witch with probability 0.5). The equilibrium is unstable in that slight deviations from the citizen’s (expected) probability of killing witches can give the government an incentive to not maintain regulation in iterated play. Specifically, if the government had reasons to believe that citizens will refrain from killing witches with probability greater than 0.5, the government would relax regulation. In such a circumstance, the citizen is better off switching his strategy (i.e. killing the witch). The equilibrium’s instability exposes the citizen to his least preferred outcome: the witch goes unpunished. The citizen can counter this by imposing the maximum possible penalty through playing a Grim Trigger strategy in which he refrains from witch-killing as long the government bans witchcraft, but killing witches every time after the government relaxes regulation. This ensures the witch is punished but at high cost. For the citizen, this strategy imposes the cost of witch-killing should the government refrain from regulating, and so is inferior to having the government maintain a ban without the citizen having to kill the witch. The Grim Trigger goes furthest to compel the government to maintain regulation because it imposes significant costs on the decision to relax regulation: the government will have to deal, in perpetuity, with the negative externalities of witch-killing and incur the costs of prosecuting witch-killers. Yet, even under the Grim Trigger, the government only maintains regulation if its discount rate is sufficiently low (for example, if it expects to remain in power for an extended period). In other words, even under the limiting condition that the citizen imposes the maximum cost he can in iterated play, the government may not unconditionally maintain regulation.

Inversely, governments do not regulate witchcraft when the government has both the capacity and willingness to enforce the law against homicide, or (trivially) not enough people fear witches to kill a large number of them. This may seem unsurprising: witchcraft bans happen in weak African states where people believe in witchcraft and punish alleged witches. But the logic is consistent with patterns of organized witch-hunts in early modern Europe. Previously, the rise in witch-hunts was thought to be the result of increasing state centralization. However, more centralized states like France and England did not institute campaigns against witches (Behringer, 2004: 126–128; Hutton, 2017: 201). By contrast, relatively weaker states in the German empire reacted to the demands of peasants to persecute witches. In that area, 22,000 to 25,000 witches are estimated to have been killed between 1650 and 1700, at a rate three times higher than the European average (Roper, 2004: 17; Goodare, 2016: 177).

At the same time, for the regulation of witchcraft to dissuade citizens from killing witches, the state requires some capacity to punish witches! When a government lacks this capacity, and the citizen refrains from witch-killing, the witch will go unpunished, and this is the citizen’s least preferred outcome. In such a situation, the citizen will kill the witch regardless of what the government does. This is elaborated in Appendix 2, where I lay out alternative game structures to represent the different incentives facing governments and citizens in states of varying capacity. Here it is worth emphasizing that contemporary states, even weak ones, mobilize more resources and intervene in their citizen’s lives to a far greater degree than prior polities. Most sub-Saharan African states extract 10–20% of GDP in taxes, and spend around 5–10% of GDP on social transfers. Both numbers exceed what European states extracted and spent in 1900, which was in turn higher than historical polities: the Roman empire and France in 1700 taxed around 5% of GDP (Scheidel and Friesen, 2009: 75). Further, African citizens do see the state as having some capacity to enforce the law; in the latest Afrobarometer survey, of 32 countries, only in Namibia did a majority of citizens *not* think the police were ‘very likely’ or ‘somewhat likely’ to respond to a complaint. Across countries almost two thirds of respondents thought the police were very or somewhat likely to respond, this despite relatively high fear of crime (2016/2018, Round 7). Given this, we would expect that allegations of ‘occult violence’ would be taken to the government. This contrasts with the early 20th century, when local authorities like chiefs managed conflicts over witchcraft (Evans-Prichard, 1935; Schapera, 1969; Waller, 2003). Even though chiefs are still active in local dispute resolution, their power relative to the government has diminished in most places. Chiefs no longer collect taxes, for example, which they did in the precolonial period (Baldwin, 2016: 33).

The model is specific because both players condition their strategies on what the other does, but generalizing it clarifies the central claim that a government that cannot enforce law and order might substitute regulation for that enforcement. In the model, the citizen wants the witch to be punished but conditions his behavior on what the government does, killing the witch only when the government does not ban witchcraft, and refraining from witch-killing when the government does ban witchcraft. The government wants to avoid banning witchcraft but conditions its behavior on what the citizen does, banning witchcraft only when the citizen kills the witch and not banning witchcraft if the citizen refrains from killing the witch. There are three other combinations: the citizen always kills witches and the government conditions its actions on what the citizen does; the citizen conditions his behavior on what the government does and the government never bans witchcraft; the citizen always kills the witch and the government never bans witchcraft. As Table 1 below summarizes, there is always an equilibrium in pure strategies in these three other combinations, in contrast to the model (Appendix 2 provides more elaboration).

**Table 1.** Alternative combinations of strategies.

Citizen	Government	Equilibrium
Conditions behavior	Conditions behavior	No pure-strategy equilibrium
Always kills	Conditions behavior	Kill-ban
Conditions behavior	Never bans	Kill-don’t ban
Always kills	Never bans	Kill-don’t ban

This has substantive implications. First, governments unable to enforce prohibitions on verifiable crimes, like murder, might instead regulate unverifiable actions, like witchcraft, which are purported to lead citizens to engage in verifiable crimes, of which more in the penultimate section. Second, the mixed strategy suggests that citizens can compel such regulation without incurring high costs: killing witches some of the time can compel the government to ban witchcraft.

Conversely, when a government always prefers not to ban witchcraft, the citizen always kills the witch, even if he would prefer the government to ban witchcraft to killing the witch himself. To prevent this, the government must enforce the law against murder. Regulation imposes costs – the government punishes witches – but abjuring regulation also imposes costs – the government must tolerate disorder or punish witch-killers. This suggests that governments committed to not expanding regulation and maintain order will need to invest greater resources to punish citizens. The absence of regulation, then, does not follow from lower state capacity; it may in fact require higher capacity, which will be developed in the penultimate section.

To summarize, the model suggests that, when witch-killing is high, regulating witchcraft may be preferred to enforcing basic laws because it is less costly. But over the long run, because the government may relax regulation when citizens refrain from harming witches, the improvement in public order may be short-lived. However, the state must have some capacity to punish witches (as most states in Africa do), otherwise citizens will always harm witches. Finally, because it imposes costs on witches, whose ‘crimes’ are unverifiable, such regulation is normatively troubling.

## Regulating from a position of weakness

In this section, I turn to the conditions under which the regulation of witchcraft might lead to more salutary outcomes. The model suggested that the regulation of witchcraft imposes costs on individuals whose ‘crimes’ are difficult to verify, and that the regulation cannot be sustained in iterated play because the government has incentives to renege once the citizen refrains from harming witches. At the same time, the case for regulation is simply that the cost of not regulating, in terms of the deterioration of law and order and the legitimacy of the government, is higher than the cost of regulation plus the cost to the alleged witch. Put another way, it behooves us to take the regulation of witchcraft seriously as a rational response to a problem of order. To this end, I analyze why governments might continue to regulate witchcraft even after the citizens refrain from harming witches. This leads to a different conclusion than suggested by the literature on neo-patrimonialism; in contrast to that literature, I suggest that this sort of regulation may not always be inefficient.

The problem the model identifies is that the maintenance of regulation by the government inflicts costs on the government and alleged witches, while the benefit accrues to those who would harm witches if the government does not punish them first. Thus, the government should prefer to relax the regulation when the citizen refrains from harming witches. This is true if the government is myopic, successive games are independent of each other, and the payoffs do not change over time. But there are reasons to expect the size of the government’s payoff to grow over time if the government maintains regulation. When the government maintains the regulation, there are positive externalities for the interaction between the government and citizens outside of the model – for example, taxation or reporting crimes to the police. Citizens might feel mollified that the government respects their beliefs, as anthropologists suggest, and so begin to cooperate with the government, or at least not challenge it, in other areas. Alternatively, when citizens refrain from harming witches, the improvement in order might raise economic growth.^11^

Therefore, the payoff to even a myopic government of maintaining regulations on witchcraft, rather than first regulating and then reneging once the citizens refrain from harming witches, may exceed the cost of regulating witchcraft plus the cost imposed on the alleged witches. Of course, this continues to be to the detriment of alleged witches (and introduces the possibility of distortions like spending on traditional cures and the like). However, unlike in the restricted model above, maintenance of the regulation can improve state capacity by affecting parameters, like economic growth, outside of the model. It is understandable that African governments, faced with significant challenges to law and order of which witch-killing is part, would regulate witchcraft instead of punishing those who harm witches. But again, the real target of regulation is not witchcraft; it is the response to witchcraft, which is one of several challenges to law and order that in aggregate reduce economic growth. A government unable to satisfactorily regulate the behavior it really wishes to affect may try and affect that behavior by regulating something else.

This provides an alternative interpretation of seemingly inefficient policies common in states with lower capacity, like price controls on food. A large body of scholarship has identified such policies as based in rent-seeking or neo-patrimonialism leading to low savings rates, a stifled capitalist class, and greater likelihood of fiscal crises.^12^ In a seminal work, Bates attributed government interventions in markets to the need for African leaders to forestall urban unrest by keeping food prices low, and ascribed to them negative effects from wealth disparities to the survival of inefficient firms (1981: 59–80). On the one hand, the need to avoid unrest is similar to the motivation I have identified.

On the other hand, the inefficiency of these strategies depends on the counterfactual. The (implicit) counterfactual when neo-patrimonial or rent-seeking pressures are inferred is that in their absence, resources would be allocated based on market considerations, hence lead to greater growth (e.g. Englebert, 2000: 14).^13^ But the model presented here suggests a different counterfactual, namely, that in the absence of non-market policies, decentralized unrest might arise that would diminish growth to a potentially greater degree than would result from market inefficiencies, especially if local unrest metastasized into civil conflict. Non-market policies would, then, not necessarily be inefficient; whether or not they are is an empirical question (Mkandawire, 2015).

This suggests the expansion of state capacity in ‘weak states,’ the majority of states in the world, is occurring quite differently from how states developed in Europe and North America. On the one hand, these states face difficulties in monopolizing violence, and tax around 15–20% of GDP, compared to the OECD average of over 30%. On the other hand, contemporary ‘weak’ states extract and spend more than European states did in 1900 (Chowdhury, 2018). While states that have been democratic for long periods “without upheaval or invasion” might be expected to face the most rent-seeking pressures to regulate (Olson, 1982: 77–78), contemporary weak states are relatively young, frequently undemocratic, and many have experienced civil war. Yet, they regulate promiscuously as Appendix 1 reveals. This cannot entirely be explained by rent-seeking, as the witchcraft example suggests, nor is seemingly strange regulation necessarily inefficient, as the model suggests.

The contemporary weak state is neither a Hobbesian anarchy nor a minimalist state administering an unfettered market. It is better characterized as a state that does a lot, mostly poorly (Gupta, 2012). The model reveals part of the reason for this: regulation may be a substitute for investments in basic services like policing, and the costs for this regulation may be imposed on the wrong people. So, rather than an ideal-typical sequence where a state first establishes the monopoly of violence and property rights, then expands regulation and extends services (Besley and Persson, 2009), contemporary weak states may be doing both things at the same time, because they do not do either well.

The proliferation and power of regulation in weak states has been documented by ethnographers (Roitman, 2005: 20, 69–70). Two anthropologists noted that the “excessive disorderliness” of African states coexists with “a fetish of the rule of law, of its language and practices, its ways and means” (Comaroff and Comaroff, 2006: Chapters VII ‐ VIII). Another, trying to sell his car in Pakistan, found an error in how his chassis number was recorded in the ownership documentation. When he went to correct the error by presenting the actual car as evidence, the official refused, countering that the documentation took precedence (Hull, 2012: 30). As an Indian bureaucrat put it: “if it is not in the file, it does not exist” (Gupta, 2012: 146). Political scientists’ focus on the weakness of state institutions in much of the world should not blind us to their ubiquitous, and often expanded, reach. Anthropologists have conveyed the pervasiveness of state power, but, quite reasonably given their interests in description, have not theorized the strategies behind it. I have sought to fill this analytical lacuna. Not only do governments with low, but not minimal, capacity have incentives to promulgate regulations, but this regulation might even improve state capacity. It is an empirical question whether it does, specifically whether the improvement in law and order exceeds the costs of the regulation plus the cost to the alleged witches. In the case of witchcraft, governments regulate when they cannot enforce basic law and order to prevent citizens harming witches. If the regulation is sustained, it might improve law and order to the degree that state capacity increases. But this still raises important normative questions on who is paying the cost for such improvements.

## Who can afford not to regulate speech?

Witchcraft may appear to be a singular problem, but there are other instances where unverifiable or unverified harms can nonetheless lead to disturbances of order. I address one such: blasphemy and offensive speech more generally. The link between specific speech acts and the harm they inflict is often deemed non-existent or, at least, unverifiable, driving those harmed to protest, sometimes violently. I discuss one such example to suggest that minimal regulation of speech may require high capacity to enforce law and order.

When Salman Rushdie’s novel *The Satanic Verses* was published, two pluralistic democracies, India and Britain, faced organized protests against the book, but responded quite differently. India banned the sale of the book within 2 weeks of its UK publication after an otherwise favorable reviewer noted the book was “bound to trigger an avalanche of protests from the ramparts” and two MP’s demanded a ban (quoted in Pipes, 1990: 19). The ban was imposed by the Finance Ministry, under the Customs Act regulating imports, not under the provision of the Penal Code that punishes acts outraging religious sensibilities (Appignanesi and Maitland, 1990: 40). Rushdie (1988) himself noted that his book had been banned despite being deemed not to outrage religious sentiments but because it might have been distorted by unscrupulous actors. Just like the regulation of witchcraft is targeted at the reaction to witchcraft, the object of regulation in this instance was the reaction to the book, not the book itself.

The British government, by contrast, did not ban the book. Home Secretary Douglas Hurd stated “it is not the job of British ministers to go about condemning books” and Prime Minister Thatcher wrote to Muslim leaders that “there were no grounds” for a ban (Pipes, 1990: 21–22). One might attribute the variation in government reactions to differences in political culture and the role of religion in public life: one of Rushdie’s Indian critics, the MP Syed Shahabuddin, stressed that Indians were a religious people in contrast to their Western counterparts (Appignanesi and Maitland, 1990: 38). But it was equally the case that the Indian government faced greater difficulties in suppressing protests and unrest. Concerns about public order may have motivated individuals otherwise not concerned with religious sensibilities like the writer (and noted atheist) Khushwant Singh to recommend the press not publish the book in India. It follows that a pluralistic political culture is not sufficient for a lack of restrictions on speech. *Ex ante* restrictions on speech may occur in a pluralistic political culture where the capacity for policing is low.

India’s ban on *The Satanic Verses*, while the most prominent, is not anomalous. More than 50 books have been banned in India since 1947 by the national government or state governments.^14^ These fall into four categories: those that defame prominent leaders; those that are deemed to critically misrepresent India or its foreign policy; those that outrage religious sentiments; and those, like *The Satanic Verses*, that have caused or are expected to cause unrest. The first two categories dominated the list of banned books in the four decades after independence, during which all bans were decreed by the central government. Subsequently, the number in the last two categories has risen, with state governments promulgating a third of the bans. At least five of these bans have been struck down by courts, including the Supreme Court, which have charged the government with the responsibility to prevent unrest or protect writers. In the UK, by contrast, very few books have been banned in the 20th century, and none since 1990. The modal reason for bans in the UK is obscenity (e.g. *Ulysses*, *Lolita*). This pattern fits with changing mores in Britain, but, at the same time, no book has been banned because it caused or was expected to cause unrest.

The case of *The Satanic Verses* fits the broader pattern of book bans in India and the UK. As citizens have threatened unrest, national and state governments in India have responded by banning books, and occasionally, been chastised by courts to prevent unrest rather than ban books. The UK government has not. While this is suggestive rather than dispositive, it does support the implications of the model. The relationship between regulation and state capacity is not straightforward. States with high capacity to supply regulation may, for that reason, be able to refuse some demands for regulation, even as they accede to other rent-seeking demands. Conversely, states that respond to demands for regulation may be doing so because they lack the capacity to supply basic law and order.

## Conclusion

In 2016, a player in the Rwandan soccer league was accused of an act of witchcraft during a match. This led to a fracas. Afterwards, the regulatory body for Rwandan soccer, FERWAFA, announced that competitors engaging in witchcraft would face fines and suspensions. In explanation, a senior FERWAFA official said, “since there is no scientific way to prove the use of witchcraft, these measures will be based upon reports from match officials and anything that is deemed to incite witchcraft will be put under consideration … [T]here is nowhere in the world where [witchcraft] has been proven that it can influence the outcome of a game … However, with the violence between players because of allegations that one team is using it, we have decided to enact laws” (quoted in Payne, 2016). By contrast, in 2018, the Parliament of Canada removed Section 365 of the Criminal Code, dating from 1892, which had previously proscribed the pretence of witchcraft to defraud individuals. An MP explained, “Canadians are far better served by a criminal code that is focused on conduct that actually causes harms or risks causing harms to Canadians” (Parliament of Canada, 2017).

These vignettes encapsulate the problem witchcraft poses to governments. The harms of witchcraft cannot be verified, yet witchcraft leads people to act in ways that compromise public order. As such, witchcraft presents a limiting case for regulation: no organized interest can benefit from its regulation; and governments that regulate it have fewer resources to regulate in general. I have suggested that a state regulates such an activity when it is unwilling or unable to incur the cost of fulfilling its basic functions – in this case to prosecute those who would harm witches – and instead promulgates potentially normatively troubling regulation – in this case to punish alleged witches – as a substitute for those functions. Such ‘regulating from a position of weakness’ can be expected to emerge in most states in the world.

Ironically, the lack of the power of the state can lead to an increase in its writ, even into occult or ‘invisible’ realms (West, 2005). Odd as it may seem, this may be better than the alternative, which is (greater) harm inflicted on witches and a disruption of law and order. The regulation of witchcraft allows us to understand a peculiar feature of many contemporary states: proliferating efforts at regulating many aspects of everyday life, even those, like witchcraft, that would appear to be beyond regulation. I have contended we need to augment standard public choice arguments to better understand this process.

## Supplemental Material

Supplemental Material - Regulation and state capacity Click here for additional data file. Supplemental Material for Regulation and state capacity by Arjun Chowdhury in Rationality and Society

## Notes

1. My emphasis on the unverifiable nature of witchcraft will appear hermeneutically unsympathetic to those who fear witches or who have been threatened by unscrupulous individuals threatening ‘occult violence.’ In fact, I am taking very seriously the *effects* of that fear, which drives some to harm witches due to sincere beliefs in witchcraft, or creates a permissive environment for those who do not believe in witchcraft to harm witches for personal gain. To analyze this phenomenon, it is not necessary to believe in witchcraft, it is sufficient to accept that others act as if they do.

2. The difference in responses is because the countries surveyed and the question wording varied.

3. I thank Michael Weaver for these calculations.

4. A simple count of the countries with laws regulating witchcraft on the books (or not) is uninformative. Some governments punish witches in the absence of laws against witchcraft; some countries have laws on the books but minimal evidence that witches are prosecuted.

5. E.g. Ethiopia and South Africa are relatively capable states, but less than 20% of Ethiopians report they believe in witchcraft while a majority of South Africans do, so it is unsurprising that only the latter tries to regulate witchcraft (Pew, 2010: 178).

6. Fisiy surveyed forty such cases and, entirely predictably, found “no consistent pattern” to the judgments.

7. The R package, *hop*, used to generate the matrices was developed by Macartan Humphreys and is available at https://macartan.github.io/hop/

8. There are obviously verifiable harms where the government chooses not to regulate, such as with environmental pollution. But this is generally because the government claims the harm cannot be verified, and thus not regulated. By contrast, African governments regulate witchcraft despite accepting it cannot be verified.

9. The model setup is similar to Chicken, with two differences. First, unlike Chicken, the payoffs are asymmetrical. Second, more importantly, a third party, the alleged witch, incurs some of the costs imposed by certain strategies in the game.

10. Tsebelis (1990) constructed a similar model to analyze interactions between police and citizens. In that model, the police preferred to enforce speeding laws only when speeding occurred, and citizens preferred to speed when the police did not enforce. Like the model here, there was no equilibrium in pure strategies, and the only way the police (government) could prevent speeding (witch-killing) was to catch more speeders (prosecute more witches).

11. An extension is when the government, democratic or autocratic, knows it lacks the capacity to punish witch-killers, but most citizens are unaware of this. If the government does not regulate, and a few citizens harm witches with impunity, this will embolden other, non-witchcraft-related, challenges. In this scenario, the government will maintain regulation because, despite its costs, regulation prevents the revelation of the government’s underlying weakness.

12. For a review, see Mkandawire (2015).

13. Studies that test the counterfactual are rare. One is Spray and Werker (2019).

14. A crowd-sourced list is available at https://sflc.in/read-me-not-list-banned-books-india
