# Supplementary material for: Regulation and state capacity
Source: Ration Soc. 2022 Sep 30;34(4):446–68. doi: 10.1177/10434631221130850 (PMC9648979; doi:10.1177/10434631221130850)
Supplement: Supplemental Material - Regulation and state capacity [file sj-pdf-1-rss-10.1177_10434631221130850.pdf]

Appendix 1

I use three measures of state capacity – tax ratios (FIGURES A1.1-A1.4), immunization for diphtheria, pertussis, and tetanus (FIGURES A1.5-A1.8), a measure of census accuracy described below (FIGURES A1.9-A1.12) – to show that states with higher capacity do not regulate any more than states with lower capacity.

In FIGURES A1.1-A1.8, a positively sloping line indicates that states with higher capacity regulate more: we do not observe this. In FIGURES A1.9-A1.12, a negatively sloping line indicates that states with higher capacity regulate more: we do not observe this either.

The measure for regulation comes from the World Bank's 'Ease of Doing Business' index. The following data sources were used, of which the first three are from the World Bank:

Regulation: https://data.worldbank.org/indicator/IC.BUS.DFRN.XQ

Taxation: https://data.worldbank.org/indicator/GC.TAX.TOTL.GD.ZS

Immunization: https://data.worldbank.org/indicator/SH.IMM.IDPT

Censuses: Lee 2020.

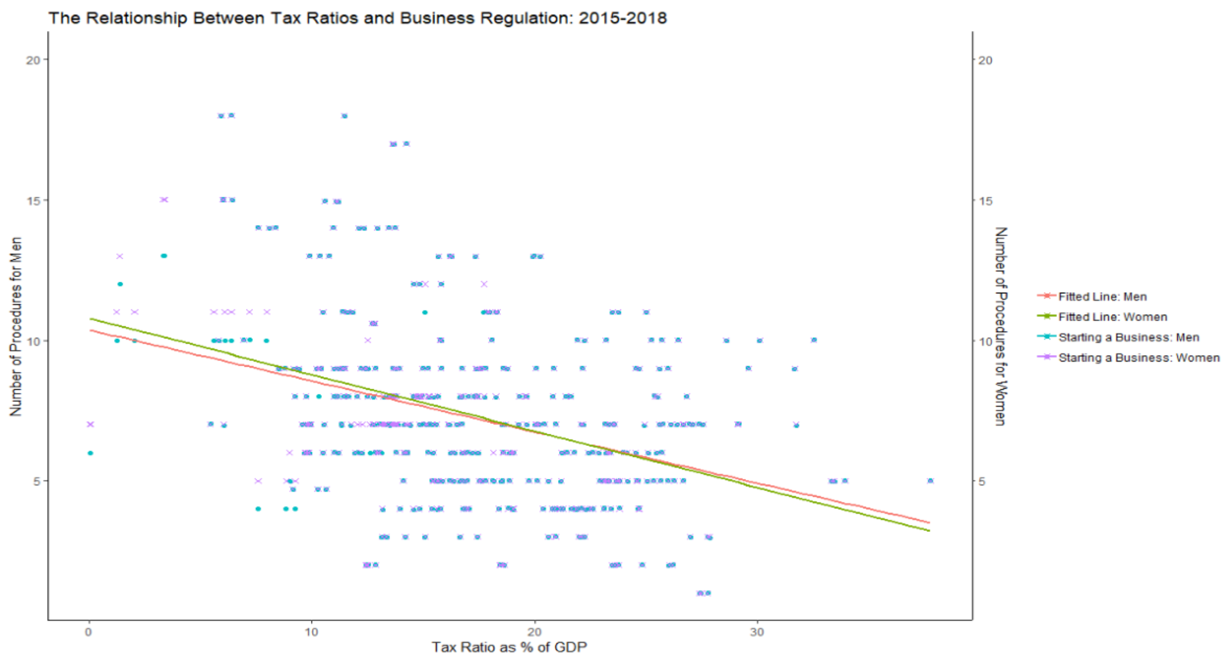

FIGURE A1.1

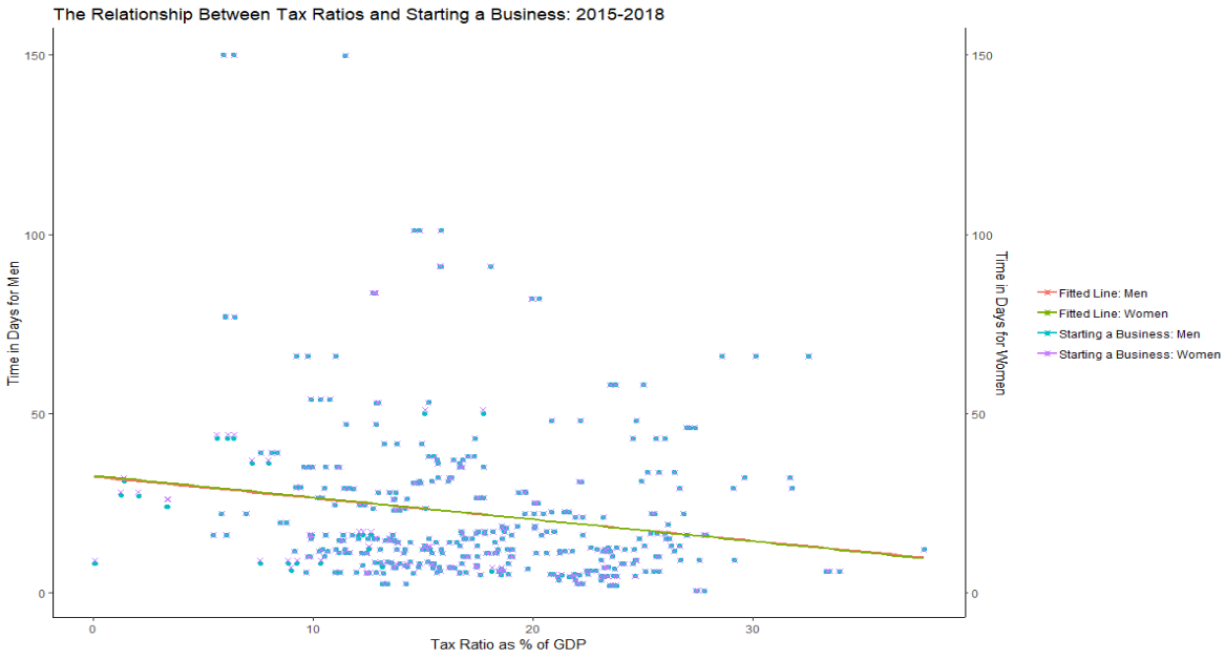

FIGURE A1.2

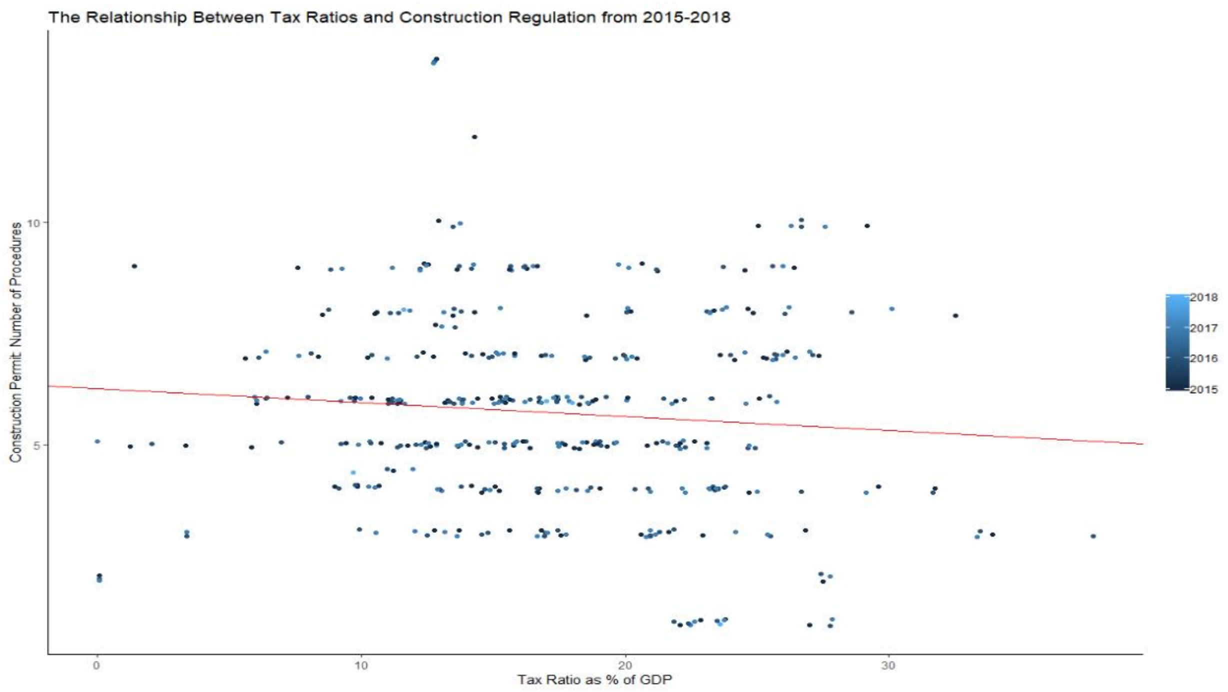

FIGURE A1.3

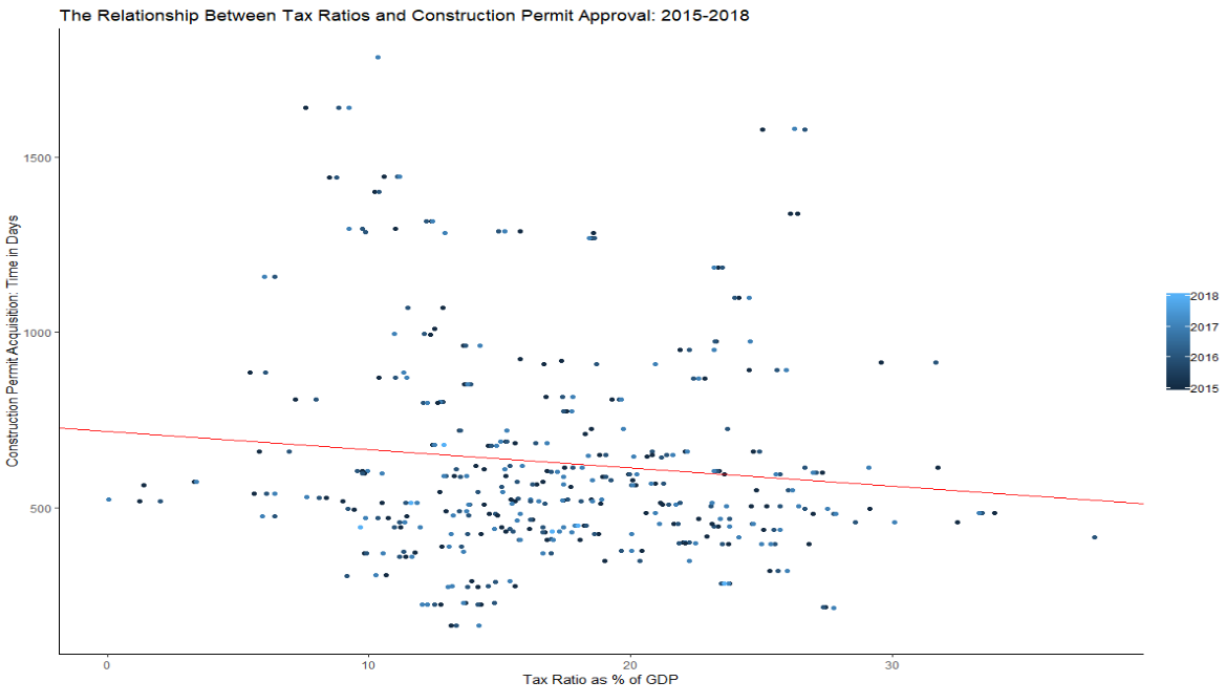

FIGURE A1.4

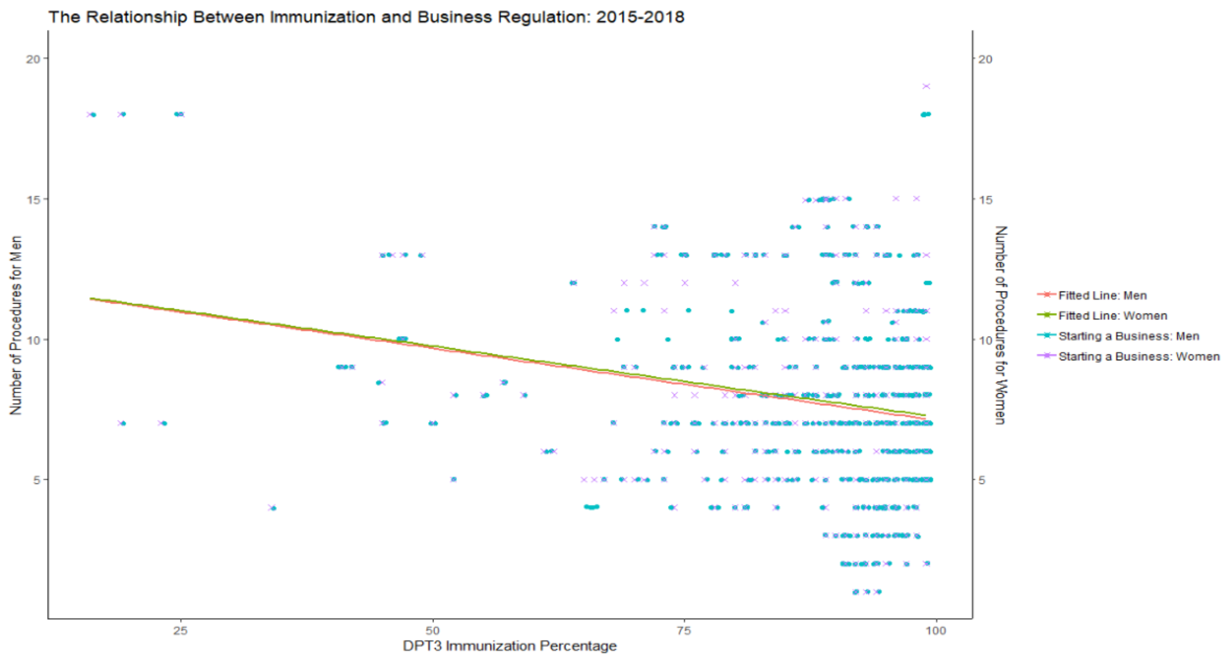

FIGURE A1.5

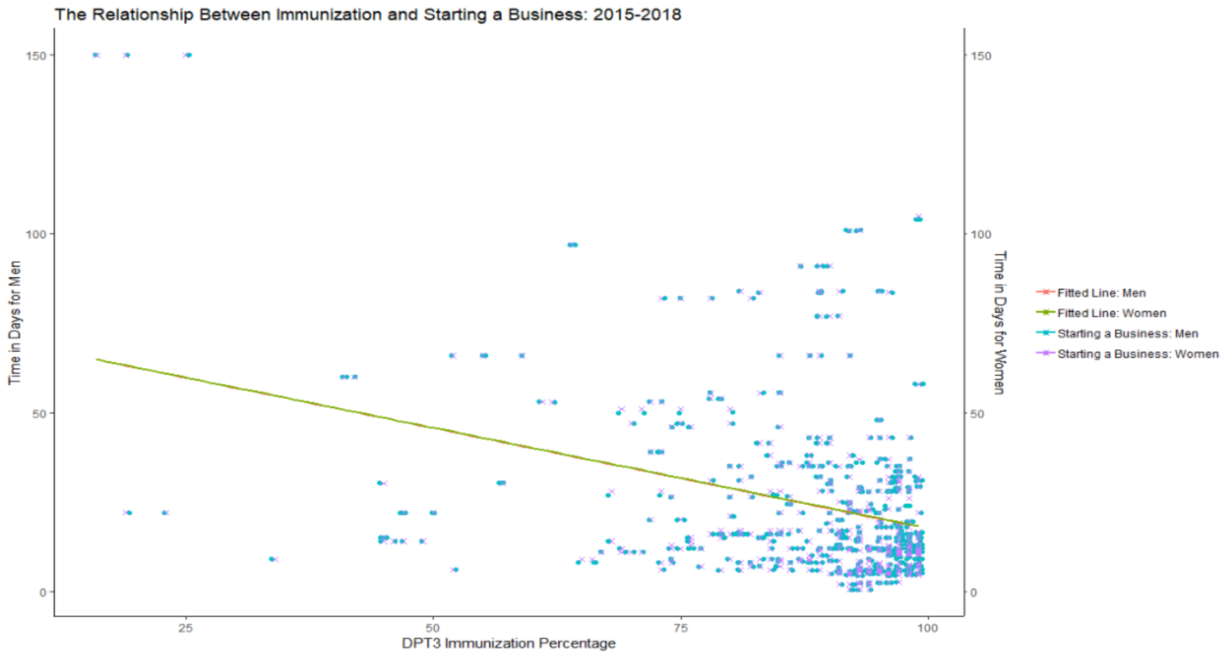

FIGURE A1.6

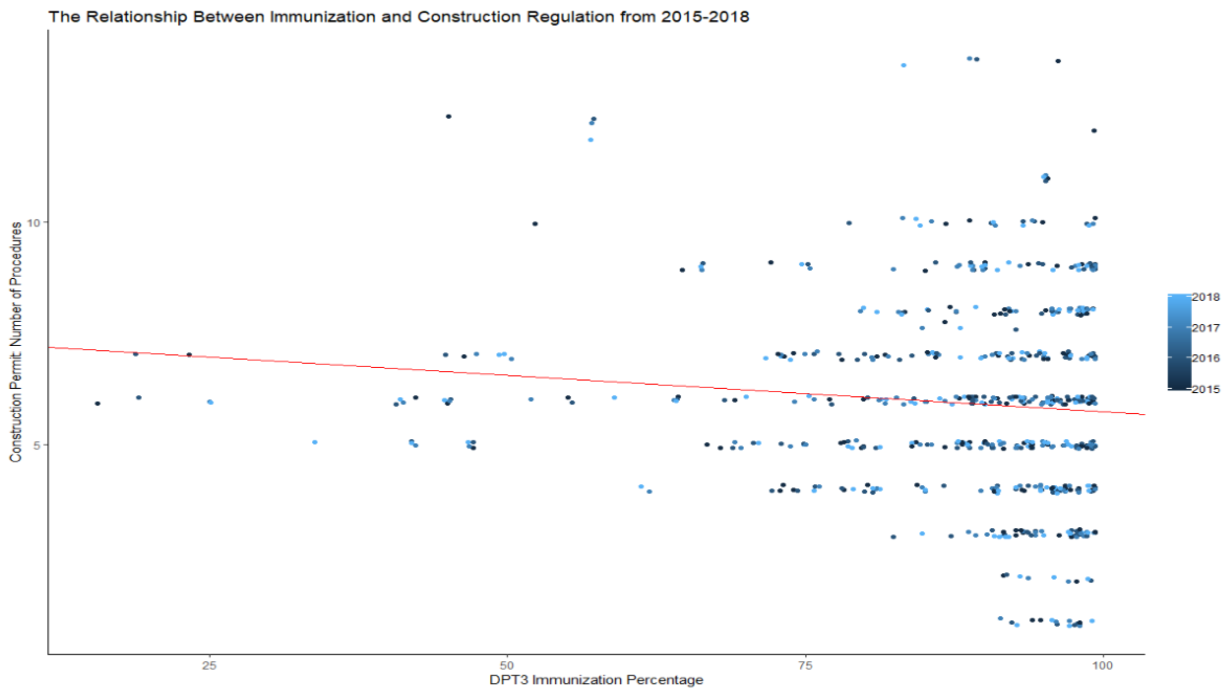

FIGURE A1.7

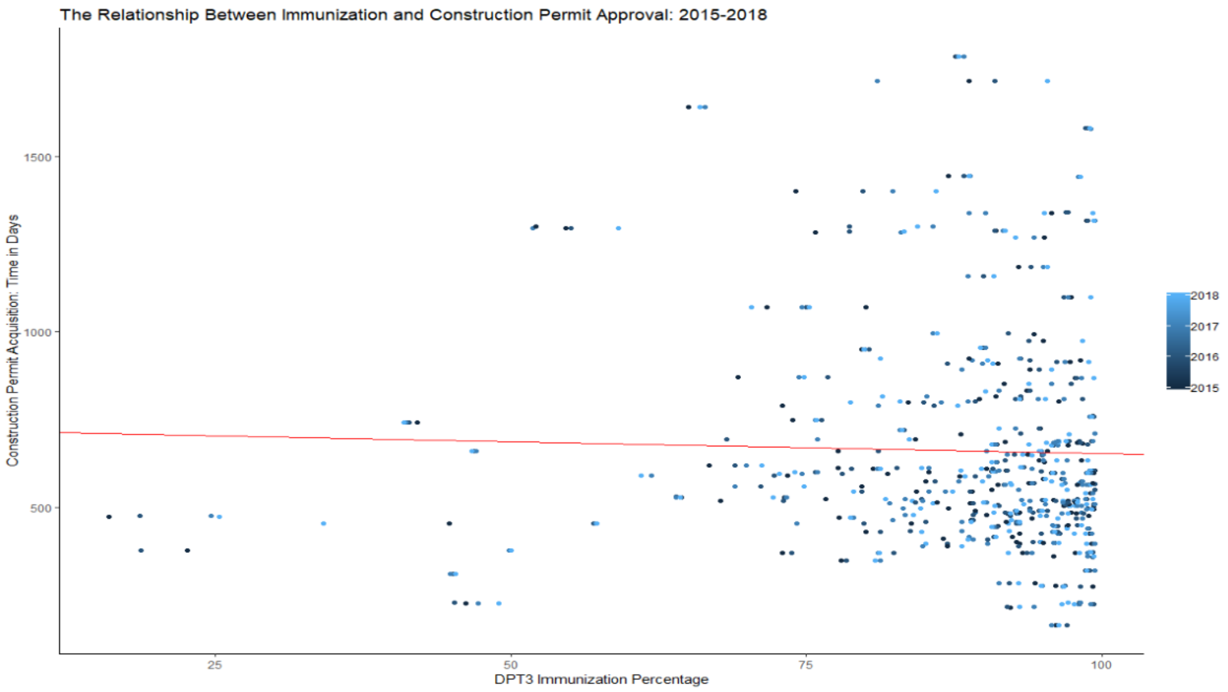

FIGURE A1.8

In the next four figures, I utilize a measure of census accuracy called the Myers Index (Lee 2020, 183-184). Akin to forensic accounting, this measures the distribution of the last digit of reported

ages. For any given decade, this distribution should be smoothly declining from 0 to 9 – e.g. there are more 60 year olds than 65 year olds, and more 65 year olds than 69 year olds – so spikes of 0's and 5's reflect guesstimation, and so inaccuracy. This inaccuracy conveys information on state capacity in two ways: it speaks to the quality or trustworthiness of the enumerators, who are state employees, and inaccurate censuses affects the delivery of state services. Higher scores mean more clustering on certain digits, hence less accurate censuses, and lower state capacity; if higher capacity states are regulating more, the slope will be negative. I use the last available Myers score for the X-axis. These scores are correlated with previously used measures of state capacity like GDP per capita.

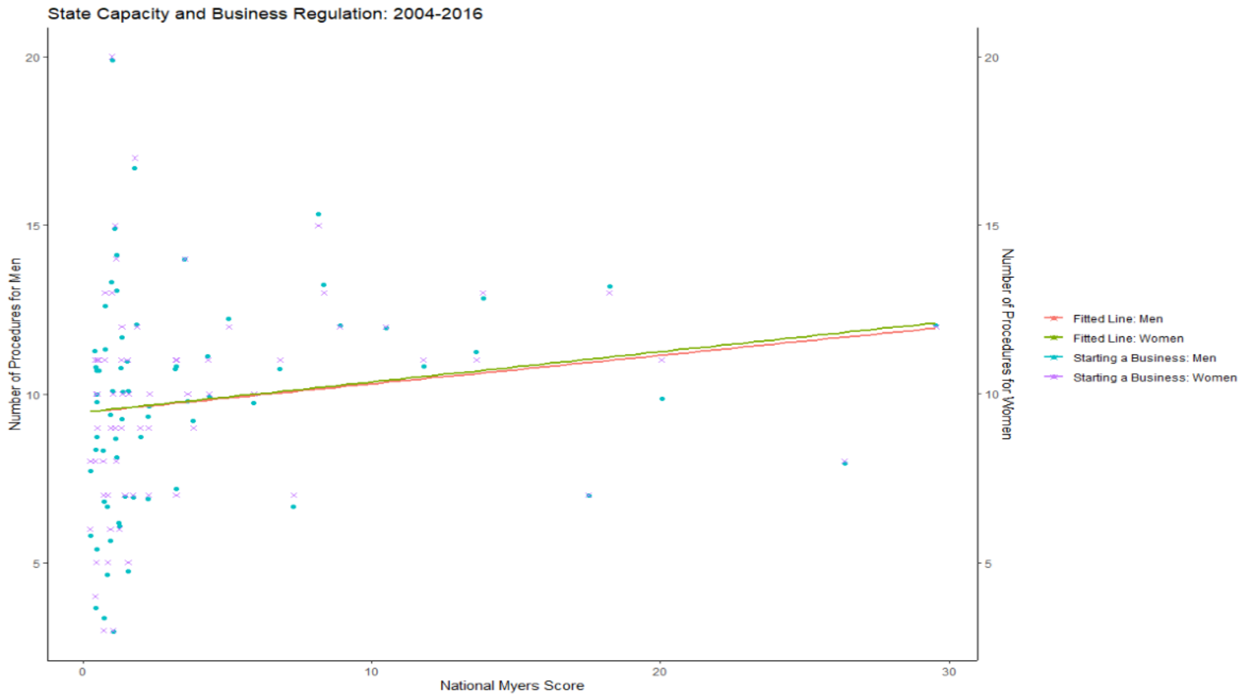

FIGURE A1.9

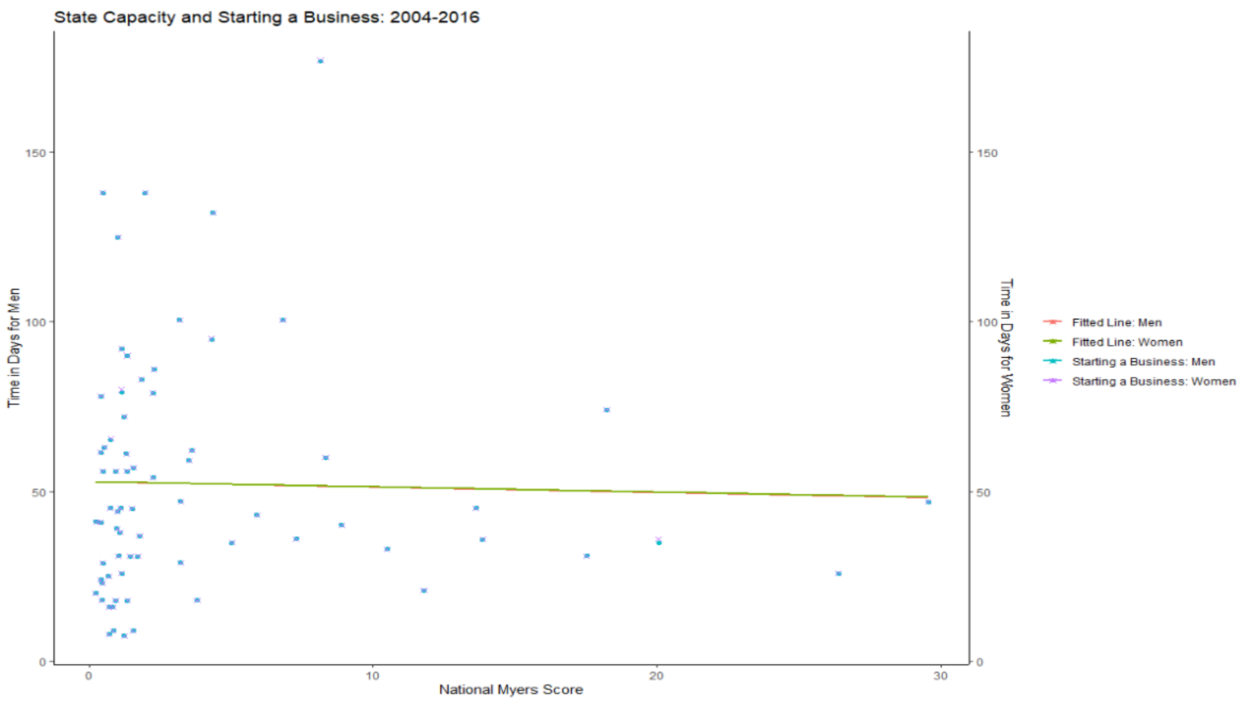
State Capacity and Starting a Business: 2004-2016
Time in Days for Men
Time in Days for Women
National Myers Score
0
50
100
150
0
10
20
30
Fitted Line: Men
Fitted Line: Women
Starting a Business: Men
Starting a Business: Women

FIGURE A1.10

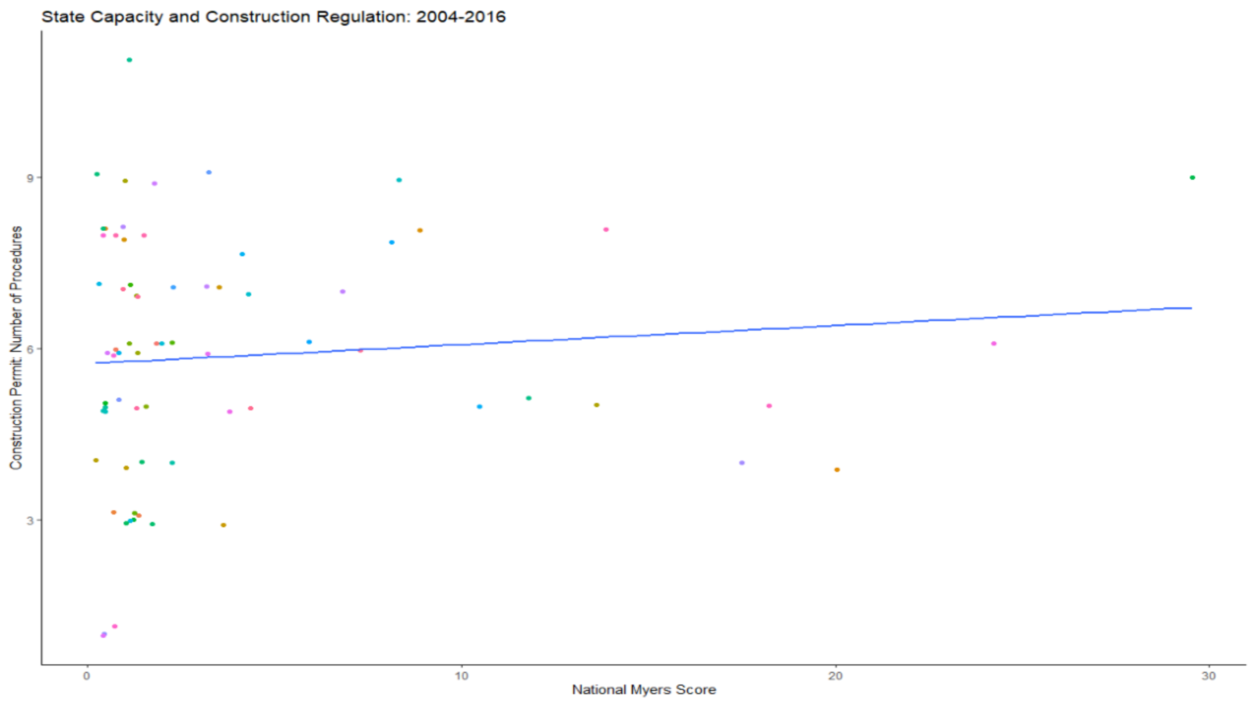
State Capacity and Construction Regulation: 2004-2016
Construction Permit: Number of Procedures
National Myers Score
3
6
9
0
10
20
30

FIGURE A1.11

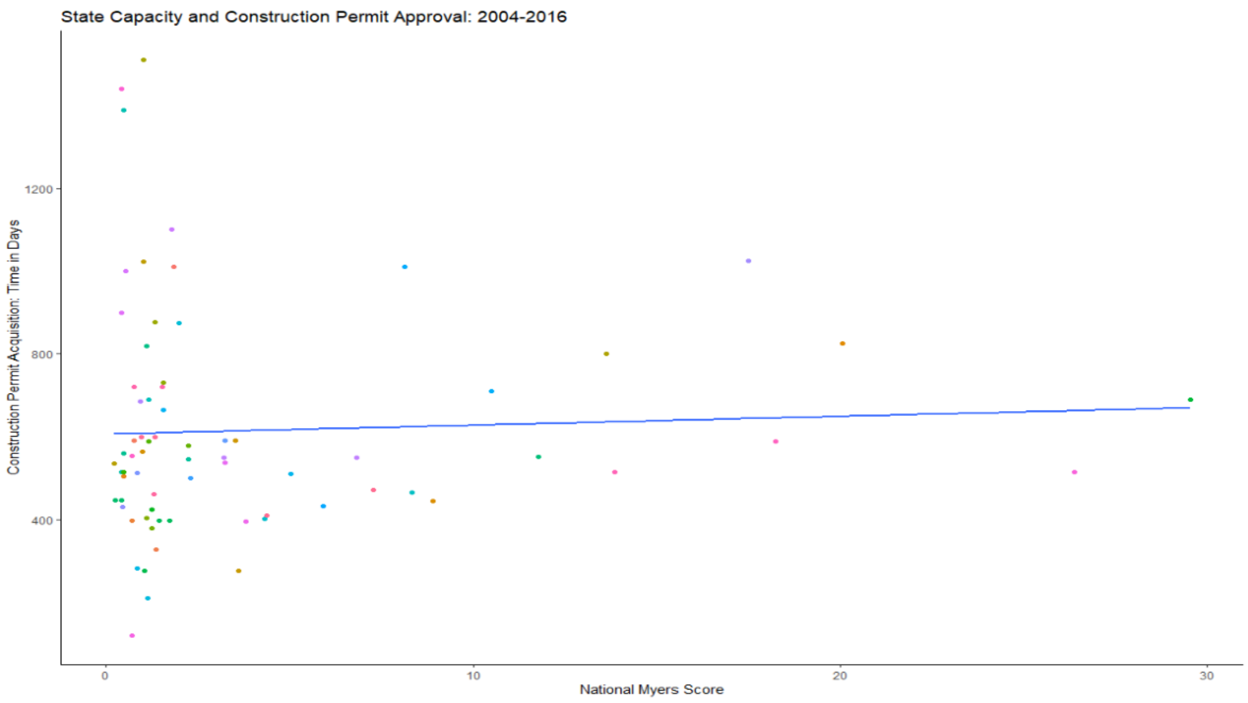

FIGURE A1.12

Appendix 2

The government's decision to regulate witchcraft depends on its capacity to punish citizens for harming witches. Three types of states can be identified: high-capacity states that can punish many witch-killers; low-capacity states that cannot punish any witch-killers; and states with intermediate capacity that can punish some witch-killers, but not many. The citizen's preferences, and so his decision, to harm the witch depends on the government's capacity to punish him for harming witches, and the government's capacity to punish witches if the government regulates witchcraft. The high-capacity and low-capacity states can be contrasted below (the arrows represent best responses, the stars represent equilibria):

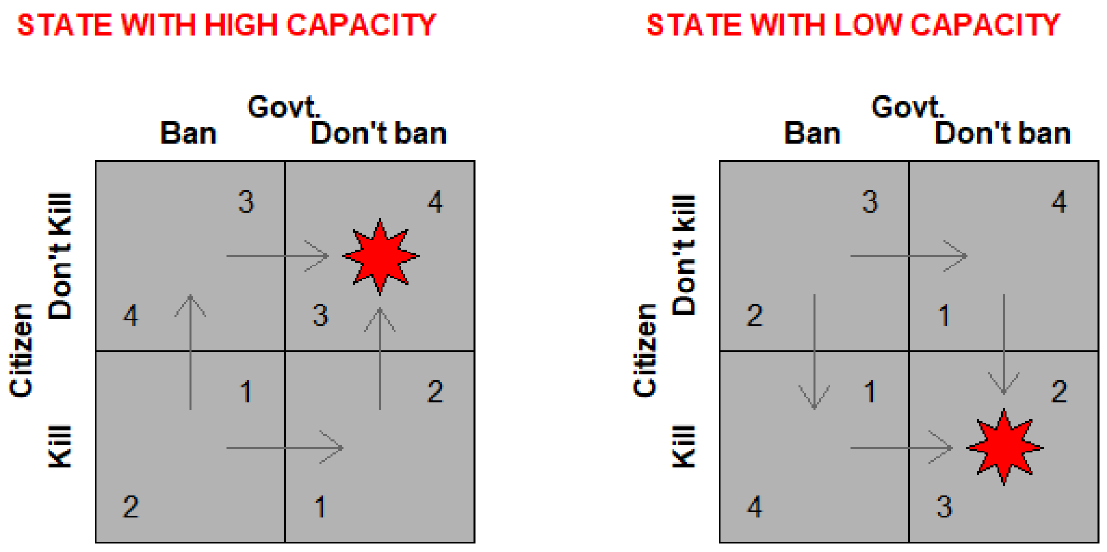

FIGURE A2.1

As FIGURE A2.1 shows, in the high-capacity state, the citizen always refrains from harming witches because of the high likelihood of punishment. By contrast, in the low-capacity state, the citizen always harms the witch because the government is unable to either punish the witch-killer or the witch.

This leaves the intermediate level of capacity: a state that is not strong enough to prosecute all witch-killers, but strong enough to prosecute some witches. Unlike in FIGURE A2.1, the citizen knows he is unlikely to be prosecuted for witch-killing, but the government, just like the ones above, has no incentive to ban witchcraft (presumably because the level of witch-killing is so low it inflicts a lower cost on the government than banning witchcraft). The citizen, like in the state with no capacity, and unlike in the state with high capacity, does choose to kill the witch. This is represented in FIGURE A2.2 below.

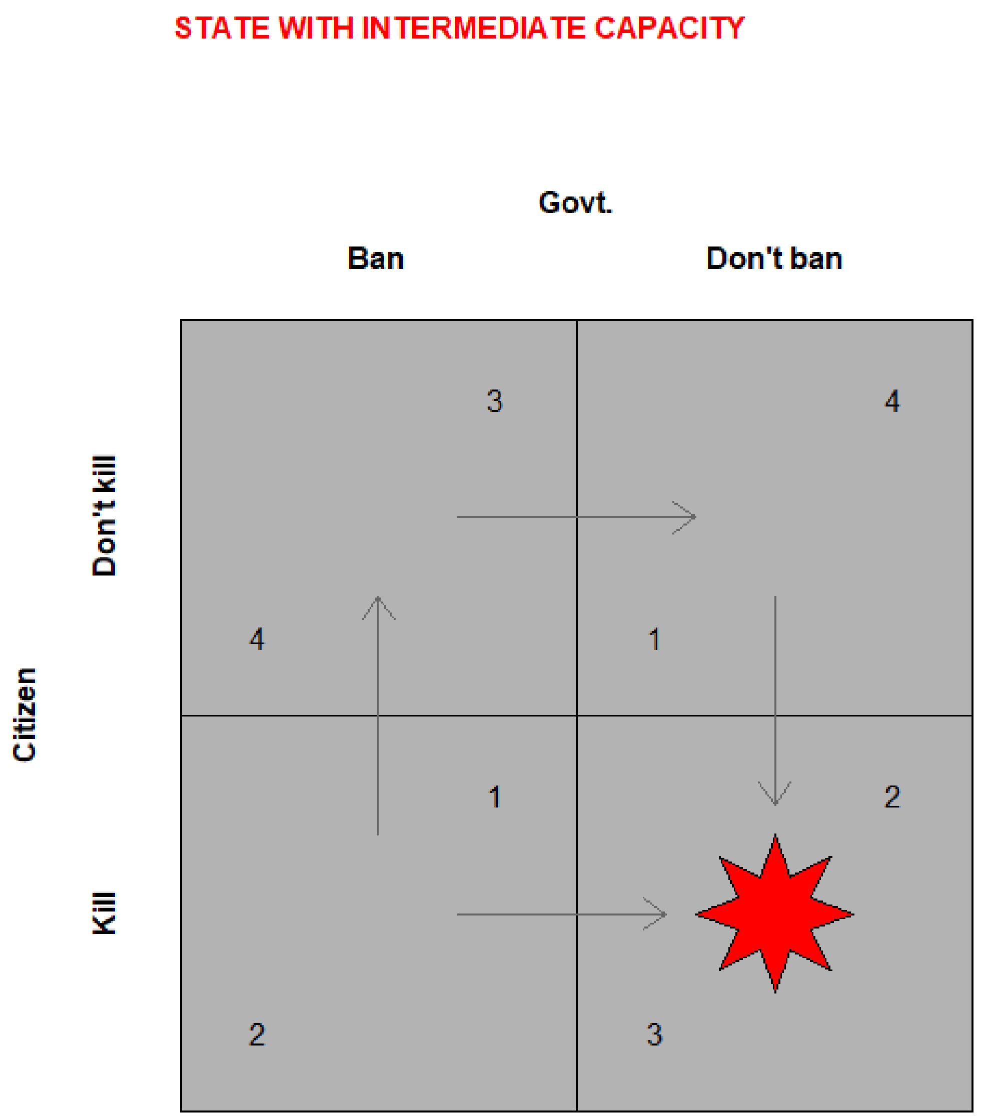

FIGURE A2.2

This situation is different from the previous two for the citizen. Unlike the high capacity state and like the low capacity state, he is likely to go free if he kills the witch. But unlike either, the citizen would prefer the government ban the witch than have to kill the witch. However, the government will not ban witchcraft, so for there to be a ban, the government's preference

ordering must change. More precisely, the cost of not banning witchcraft must exceed the cost of banning it.

This occurs when witch-killing becomes a problem of order, or more citizens kill witches than the government can punish. As witch-killing becomes a problem, the government's savings from not banning witchcraft are exceeded by the cost to public order of witch-killing. This can be accommodated by altering the matrix as in FIGURE A2.3 below, which reproduces FIGURE 1 in the text.

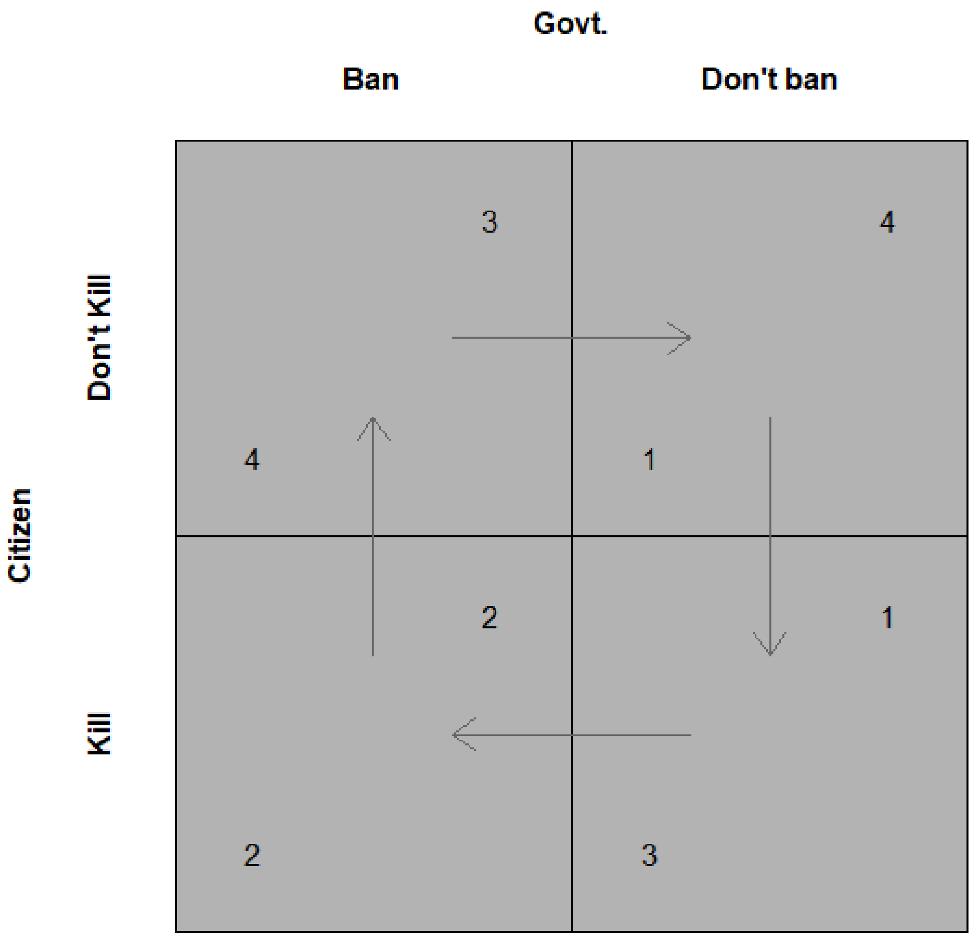

FIGURE A2.3
